# Influence of Prunus domestica gum on the release profiles of propranolol HCl floating tablets

**DOI:** 10.1371/journal.pone.0271442

**Published:** 2022-08-26

**Authors:** Salman Mehmood, Syed Muhammad Farid Hasan, Rabia Noor, Muhammad Sikandar, Syed Nadeem ul Hasan Mohani, Fauzia Israr, Syed Imran Ali, Majeed ullah, Fouzia Hassan

**Affiliations:** 1 Department of Pharmaceutics, Faculty of Pharmacy & Pharmaceutical Sciences, University of Karachi, Karachi, Pakistan; 2 Pharmacy Department, Sarhad University of Science and Information Technology, Peshawar, Pakistan; 3 Department of Pharmaceutics, Faculty of Pharmaceutical Sciences, Jinnah Sindh Medical University, Karachi, Pakistan; 4 Department of Pharmacy Practice, Faculty of Pharmacy, Ziauddin University, Karachi, Pakistan; 5 Department of Pharmacy, Kohat University of Science and Technology, Kohat, Pakistan; Bahauddin Zakariya University, PAKISTAN

## Abstract

Propranolol hydrochloride is a beta-blocker used for the management and treatment of hypertension, angina, coronary artery disease, heart failure, fibrillation, tremors, migraine etc. The objective of the present study was to design Propranolol Hydrochloride floating tablets by direct compression method and to explore the role of a new gum as a matrix former. A 2^2^ full factorial design was selected for the present study. *Prunus domestica* gum and HPMC (K4M) were used as independent variables, swelling index and drug dissolution at 12 hours as dependent variables. Formulations were subjected to pre- and post-compression tests that showed good micromeritics and buoyancy characteristics (Carr’s index 11.76%–14.00%, Hausner’s ratio 1.13°–1.16°, angle of repose 22.67°–25.21°, floating lag time 56–76 seconds, total floating time 18–25 hours and swelling index 59.87%–139.66%). The cumulative drug release in 0.1 N HCl at 12 hours was 72%–90% (p<0.05). Weibull model was found to be the best fit model (R^2^>0.99) among all other studied models. Multiple regression showed a significant effect of *Prunus domestica* gum and HPMC K4M on the swelling index and dissolution profiles of propranolol HCl (p<0.05). On the basis of better *in-vitro* performance and cost-effectiveness, formulation F4 was the best formulation. It is evident from the results that *Prunus domestica* gum possesses excellent drug release retardant potential for the floating drug delivery system and this new gum should be further explored alone or with other natural and synthetic polymers in future studies.

## 1. Introduction

Oral drug delivery is preferred over other routes of drug administration because it offers higher patient compliance, ease of administration and variety in the formulation of dosage forms [1]. However, conventional oral therapy has certain limitations such as variability in systemic drug concentration (due to unpredictable drug release from the dosage form) resulting in variations in the therapeutic index which ultimately limits an effective treatment. But these demerits can be surpassed by developing a controlled-release oral delivery system. The system is considered advantageous over conventional oral dosage forms in terms of producing a uniform and prolonged drug release, decreasing the frequency of drug dosing and reducing variation in the therapeutic index [2–4]. Besides the advantages, variations in gastrointestinal transit (GT) and gastric residence time (GRT) have been observed with the controlled drug therapy due to the physiological variabilities among the individuals.

Gastroretentive technology has been demonstrated as a solution for reducing the above problems related to controlled drug delivery from solid oral dosage forms [5]. Using the technology, the dosage form remains in the gastric region for several hours and significantly improves its GRT [6]. Various approaches have been employed to develop gastroretentive controlled release dosage forms such as polymeric bio-adhesive system, swelling and expanding system and floating or hydrodynamically balanced system [7]. Among all, the floating drug delivery system (FDDS) has been shown the most convenient and efficient method of achieving gastro retention. The system offers various advantages such as producing localized action in the stomach, increasing GRT, improving drug bioavailability, etc. [8, 9]. Moreover, due to its prolonged GI-retention, better site-specific drug delivery can be achieved [10].

Certain factors cause floating of the dosage form on the gastric media and enhance GRT such as the low density of the dosage form than gastric medium (density lower than 1 g/ml or 1.004–1.010 g/ml), the diameter of the dosage form more than 7 mm, fed state and ring or tetrahedron skeleton of the dosage form [5, 11–14]. Furthermore, the floating of a drug can be accomplished in the stomach by incorporating an aired, vacuumed or inert gas-filled floating chamber [15].

The Propranolol HCl is a non-selective beta-receptors blocker [16]. It is used in cardiovascular diseases such as coronary artery disease, heart failure, fibrillation, hypertension, angina and as well as non-cardiovascular diseases including restless leg syndrome, migraine and tremors etc [17]. It belongs to BCS Class I, absorbs completely from GIT after oral administration and attains peak plasma concentration within 1 to 3 hours. Its plasma half-life is around 3 to 6 hours making it the right choice for developing a controlled release FDDS [18]. Its solubility depends on the pH of media due to its weak basic nature, increase at lower pH values [19]. FDDS based Propranolol HCl formulations have been formulated by several investigators. Chaturvedi *et al*. developed matrix-based gas floating tablets of Propranolol HCl. HPMC K15M was used to retard the drug release. The formulation with 27.5% HPMC was found most desirable as it retarded drug release in the stomach for 12 hours [20]. In another study, Jagdale *et al*. formulated gastro retentive floating tablets (GRFT) of Propranolol HCl. These workers employed HPMC K4M, HPMC E 15 LV, Hydroxy Propyl Cellulose (HPC), xanthan gum and sodium alginate in different concentrations and evaluated the IVIV behavior of the trial formulations. It has been reported that the tablets formulated with HPC and HPMC K4M controlled the release for 18 hours while xanthan gum failed to form tablets of sufficient strength [21]. Strubing et al. developed poly (vinyl acetate) based Propranolol HCl floating tablets. The coats of Kollicoat SR 30D / Kollicoat IR were investigated from FDDS for controlling the drug release. Tablets coated with 10 mg polymer/cm^2^ SR/IR (8.5:1.5) exhibited the highest floating strength, shortest lag time and maximum floatability while the Poly Vinyl Acetate (PVA) was found suitable polymer for retarding the drug release from these tablets. The PVA controlled the drug release for 24 hours [22].

It is evident from the literature review that most of the studies have utilized synthetic and expensive excipients in the formulation of GRFT. Natural gums, obtained from plants, are non-toxic, less expensive and easily available compared to synthetic polymers [23]. They have multiple pharmaceutical applications such as binder, disintegrant, suspending and emulsifying agents. The controlled release pharmaceutical formulations have also been developed from the natural gums such as guar gum, karaya gum and xanthan gum [19, 23–25]. Similarly, *Prunus domestica* gum has been introduced recently and reported as an effective tablet binder in the formulation of oral dosage forms [26, 27]. However, to the best of our knowledge, it has not been studied for controlled release pharmaceutical application. Therefore, the present study was designed to explore this gum as a matrix former in FDDS.

## 2. Materials and methods

### 2.1. Materials

Propranolol HCl was kindly gifted by Searle Company Ltd., SITE, Karachi, Pakistan. The gum *Prunus domestica* was extracted by the method reported by Rahim et al. [28]. HPMC K4M (low-density polymer matrix), Sodium Bicarbonate (effervescent agent), Magnesium Stearate (lubricant), Talc (glidant) and Avicel PH-102 (filler) were obtained from Merck, Germany.

### 2.2 Methods

#### 2.2.1. Gum extraction

The gum was extracted from the plant *Prunus domestica* using the method reported by Rahim et al with slight modification [28]. The extracted gum was dried at 50–60°C for three hours in a tray dryer. The dried gum was hydrated (in 500ml water) for 8 hours followed by straining through muslin cloth to remove any extraneous material. It was then precipitated by adding 10mL of absolute acetone to the solution. The precipitates were separated and dried overnight at 50°C in a tray dryer. The pure gum thus obtained was stored in a tightly closed container at room temperature for future use as a matrix former in GRFT.

#### 2.2.2. Formulation of floating tablets

**Fig 1:**
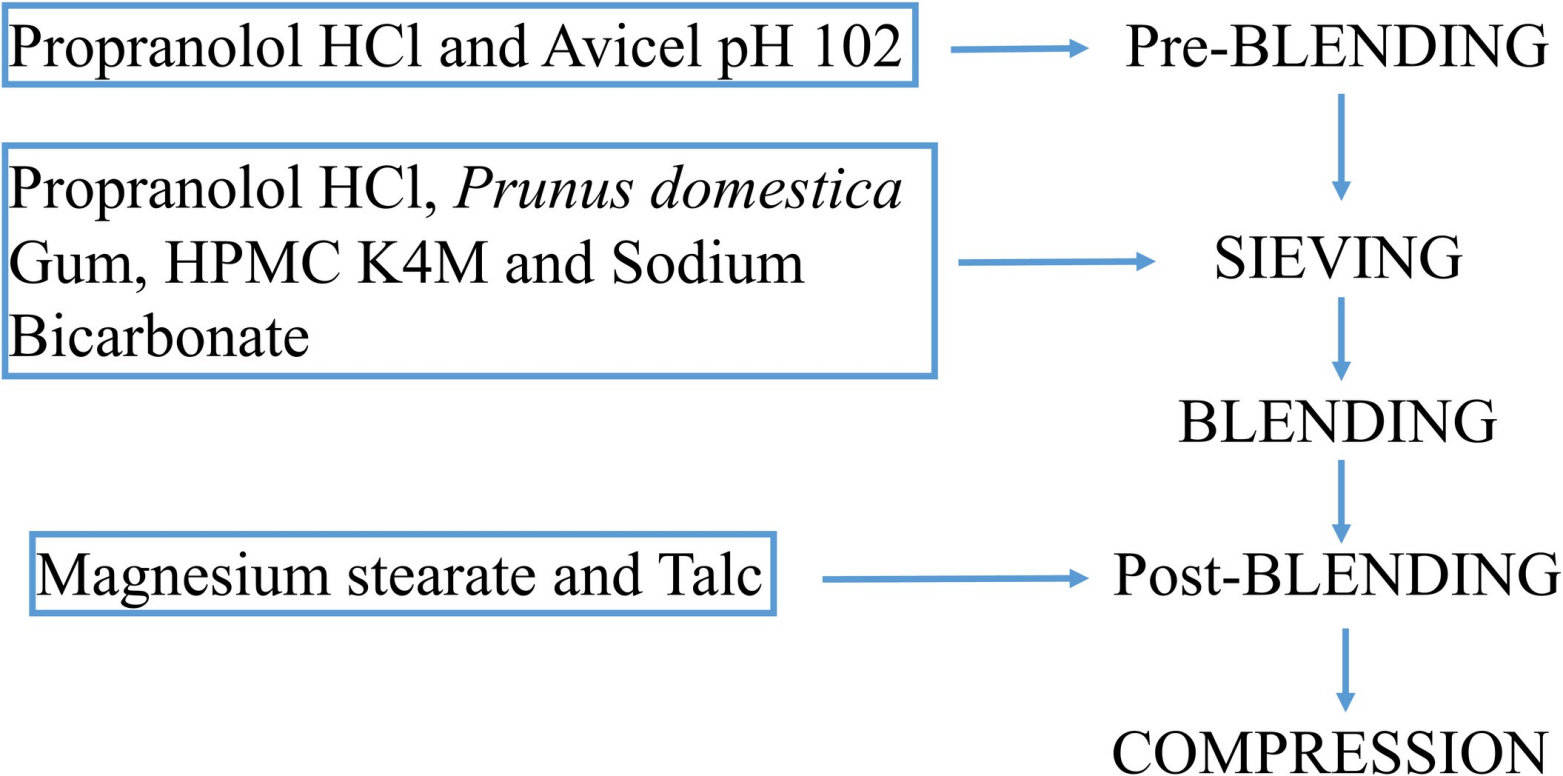
Schematic representation of direct compression of propranolol HCl floating tablets.

A 2^2^ full factorial design was employed in the current study with two factors at two levels (low and high) to determine the influence of factors, variables and their interactions at both levels. *Prunus domestica* gum and HPMC K4M were taken as independent variables while swelling index and percent drug dissolution (at 12 hours) as dependent variables (Tables 1 and 2). All the ingredients were accurately weighed in polybags. Propranolol HCl (API) and Avicel pH 102 were taken in a mortar and mixed by geometric dilution. The pre-blend was sieved through 30 mesh and transferred into a polythene bag, other formulation excipients (except magnesium stearate and talc) were also sieved through 30 mesh, added to the mixture and blended for 20 minutes to get a homogeneous mass. Finally, magnesium stearate and talc (passed through 80 mesh) were added to the polybag and the blend was further mixed for 5 minutes by tumbling. The powder blend was kept in a tightly closed container before micromeritic evaluation and compression. A single punch machine with 8 mm round punches was used for tablet compression (Fig 1). The tablet weight was set at the target weight of 250 mg/tablet.

**Table 1:** Representation of 2^2^ full factorial design.

Formulations	Independent Variables			
	coded terms		mg	
	*Prunus domestica* Gum (X_1_)	HPMC K4M (X_2_)	*Prunus domestica* Gum (X_1_)	HPMC K4M (X_2_)
**F1**	+	+	75	110
**F2**	+	–	75	60
**F3**	–	+	50	110
**F4**	–	–	50	60

**Table 2:** Formulation composition of propranolol HCl 40 mg tablets floating tablets.

S. No.	Ingredients (mg)	F1	F2	F3	F4
**1**	Propranolol HCl	40	40	40	40
**2**	*Prunus domestica* Gum	75	75	50	50
**3**	HPMC K4M	110	60	110	60
**4**	Avicel PH 102	08	58	33	83
**5**	Magnesium Stearate	1	1	1	1
**6**	Sodium Bicarbonate	15	15	15	15
**7**	Talc	1	1	1	1
**Total Compression Weight (mg/tab)**		**250**	**250**	**250**	**250**

#### 2.2.3. Micromeritic properties of the floating swellable tablets

Bulk and tapped densities were determined by taking approximately 2 g powder blend in a 100mL graduated cylinder. Bulk volume was noted by tapping the cylinder twice while tapped volume was noted until no further change in volume was observed after the continuous tapping. The respective densities were calculated by -1 and -2 [29]. The data thus obtained were used for the calculation of Carr’s Index (CI) and Hausner’s Ratio (HR) by -3 and -4 [30]. The angle of repose was calculated by the fixed height method. A funnel was fixed on a stand at a height of 1cm above the hard flat surface. The powder was poured through the funnel and a cone of the powder blend was formed on a paper. The height of the cone and radius of the powder blend were noted and the angle of repose was calculated by -5 [29].


$$\rho_{bulk}=M/V_{b}$$ (1)

$$\rho_{tapped}=M/V_{t}$$ (2)

$$\text{CI}=V_{b}-V_{t}/V_{b}*100$$ (3)

$$\text{HR}=V_{o}/V_{f}$$ (4)

tanθ=h/r (5)

#### 2.2.4. Physical tests

Weight variation, thickness, diameter, hardness and friability tests were conducted. Twenty tablets were selected randomly and the weight of individual tablets was noted using a weighing balance. The thickness, diameter and hardness of 10 of the selected tablets were measured using Vernier Caliper and Pfizer hardness tester. Upper and lower control limits along with mean and standard deviation (SD) were calculated. The friability test was performed on 20 randomly selected tablets for 4 minutes at the rotational speed of 25 rpm (100 rotations) in a Roche Friabilator. The tablets were shaken to remove the excess powder and cleaned with a soft brush. The de-dusted tablets were weighed and their friability was calculated by -6.


$$\%\text{F}=W_{i}-W_{f}/W_{i}*100$$ (6)

#### 2.2.5. In-vitro buoyancy studies

The floating Lag Time (FLT) and Total Floating Time (TFT) of each formulation were determined. Five tablets were randomly taken in a separate beaker, 500 mL 0.1 N HCl solution. The FLT was noted after the tablets started to rise on the surface while the TFT was noted by determining the time tablets remained floating on the surface [31]. Similarly, the Swelling Index (SI) was determined on 3 tablets selected individually from each of the formulations. The tablets were weighed (W_i_) and poured into a beaker containing a 100 mL solution of 0.1 N HCl maintained at 37°C. After certain time tablets were removed from the beaker and their surface was cleaned with tissue paper to remove the excess liquid. The final weight was determined, and SI was calculated by -7 [32].


$$\%\text{SI}=W_{f}-W_{i}/W_{i}*100$$ (7)

#### 2.2.6. Assay of the drug

Ten tablets were taken randomly and finely powdered in a mortar with a pestle. A sample equal to 40mg of crushed tablets was taken into a 50ml volumetric flask, 20 ml of 0.1N HCl was added and shaken vigorously till dissolved. The final volume was made up to the mark with the same solvent. The absorbance of the sample was measured by a UV double beam spectrophotometer at a 289 nm wavelength [5].

#### 2.2.7. In-vitro dissolution study

The dissolution was performed in a USP type-II dissolution apparatus using 900ml of 0.1N HCl as a dissolution medium. The temperature of the media was maintained at 37°C±0.5°C. Around 5ml aliquot was withdrawn at specified time intervals i.e., 0.5, 1, 2, 4, 6, 8, 10 and 12 hours from the vessel and media was replenished with the same volume to maintain the sink condition. The samples were filtered and absorbance was taken at 289 nm by UV spectrophotometer [5]. The percent drug dissolution was calculated by -8.


$$\%\text{DD}=A_{s}-A_{st}$$ (8)

The *in-vitro* drug release data were fitted into different kinetic models such as zero order, first order, Higuchi, Korsmeyer Peppas, Hixon Crowell and Weibull models to determine the mechanism of drug release. The data were also compared by similarity (*f1*) and dissimilarity (*f2)* factors. Moreover, multiple regression (MR), is considered to be an effective tool for generating equations that predict the relationship between the independent and dependent variables [33] One-way ANOVA was applied to the responses to find the relationship between the two variables.

## 3. Results and discussion

### 3.1. Micromeritics of trial formulations

Bulk and Tapped densities ranged from 0.43 to 0.46 g/cm^3^and 0.50 to 0.53 g/cm^3^ respectively. These values are closer to each other which indicates non-significant inter-particulate interactions among the formulations excipients good packing characteristics, important for storage, flow and compaction of the powder during manufacturing [34]. These results were further verified by corresponding Carr’s index and Hausner’sratio which were 11.76 to 14.00% and 1.133 to 1.162% respectively. To ascertain flowability, the angle of repose was determined that ranged from 22.67 to 25.21° respectively. This data indicates excellent flow and good compressibility characteristic of the powders blends (Table 3) [35, 36]. This ensured minimum batch-to-batch variation and uniform content distribution of all formulation blends which is a prerequisite for satisfactory compression [37].

**Table 3:** Pre-compression parameters of propranolol HCl floating formulations.

Formulations	Bulk Density (g/cm^3^)	Tapped Density (g/cm^3^)	Carr’s Index (%)	Hausner Ratio	*Angle of Repose (θ)	Remarks
**F1**	0.43	0.50	14.00	1.162	23.45	Good
**F2**	0.45	0.51	11.76	1.133	22.67	Good
**F3**	0.46	0.53	13.20	1.152	24.93	Good
**F4**	0.44	0.50	12.00	1.136	25.21	Good

* Angle of repose showed excellent flow property

### 3.2. Characteristics of the tablets

All formulations were compressed without any tableting defects. The average weight of tablets ranged from 249.95±2.39 mg to 250.85±3.00 mg. The hardness ranged from 7.77±0.45 kg/cm^2^ to 8.84±0.57 kg/cm^2^. The hardness was found favorable for the floating tablets as evident from a previous study [38]. The percentage friability was found within the pharmacopeia limit (< 1%) in all trial formulations. All results followed the official standards and were essential for robust tableting characteristics. The trial formulations showed API content within the range (97–99%, p>0.05) and thus complied with BP specifications that require 90–110% API in the composite samples (Table 4). This indicated appropriate blending and uniform random mixing of all the formulation ingredients.

**Table 4:** Physicochemical characteristic of propranolol HCl floating tablets.

Formulations	Weight variation (mg)	Hardness (N)	Friability (%)	Diameter (mm)	Thickness (mm)	Assay (%)	Floating Lag Time (sec)	Total Floating Time (hrs)
**F1**	250.65±2.58	8.84±0.57	0.079±0.002	7.86±0.03	3.75±0.01	97.64±0.94	76±8.75	25±2.24
**F2**	250.85±3.00	8.61±0.52	0.076±0.000	7.91±0.03	3.75±0.01	98.19±1.03	56±5.92	18±1.58
**F3**	249.95±2.39	8.10±0.46	0.021±0.002	7.90±0.03	3.76±0.01	100.23±1.96	63±8.06	20±2.55
**F4**	250.30±2.03	7.77±0.45	0.014±0.001	7.95±0.03	3.76±0.01	99.73±0.62	60±5.78	21±2.35

### 3.3. In-vitro buoyancy studies

#### 3.3.1. FLT and TFT

**Fig 2:**
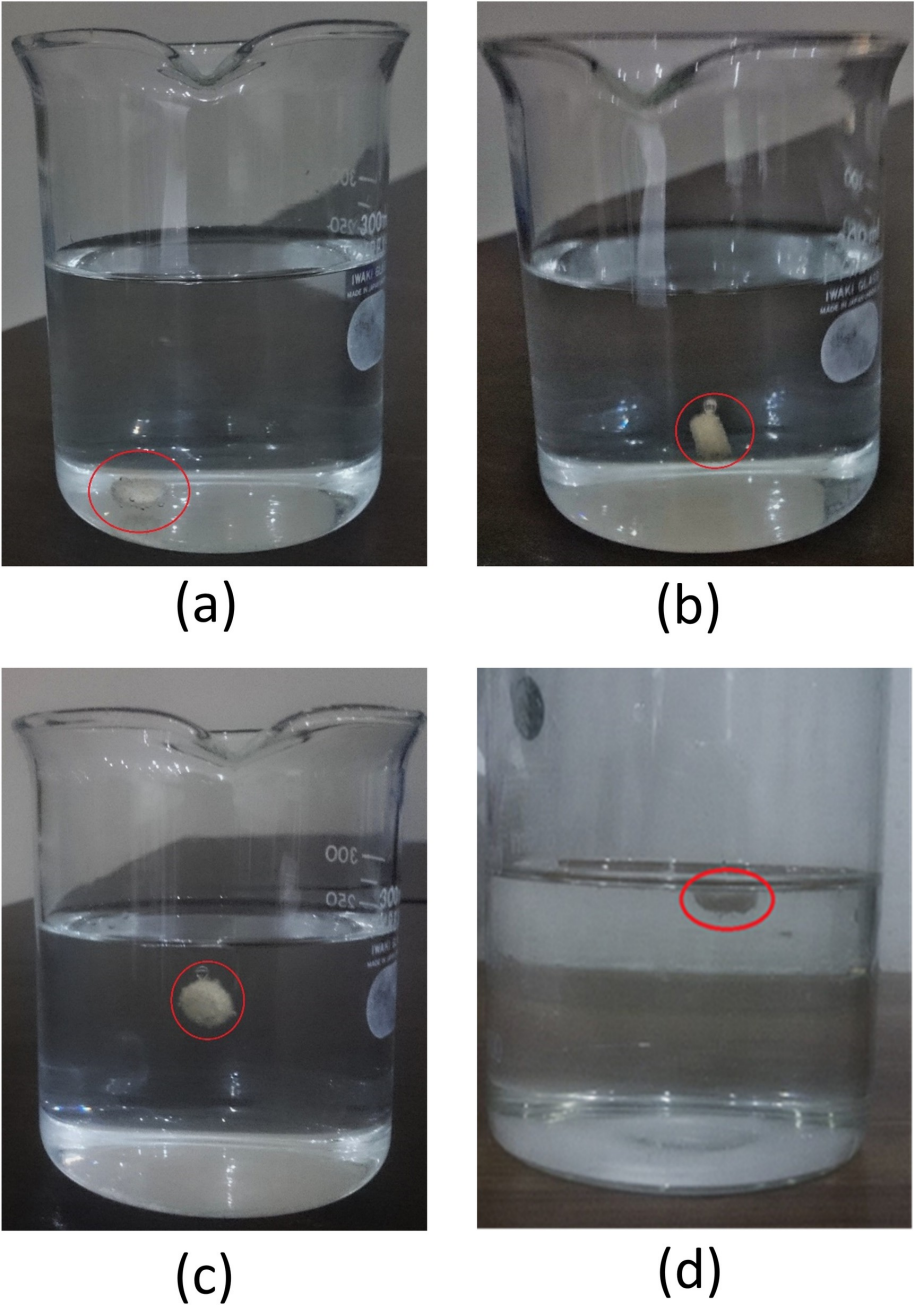
Invitro buoyancy studies of propranolol HCl (a) Propranolol HCl tablets behavior immediately after immersion to 0.1N HCl, (b) after a few seconds of immersion, (c) after one minute of immersion, (d) after 12 hours of immersion.

The FLT ranged from 56±5.92 to 76±8.75 seconds (Table 4). The sodium bicarbonate was used as a gas-generating agent in present formulations. It has been reported that it reacts with water and generates carbon dioxide gas (Fig 2). The resulting gas entraps within the polymer matrices and ultimately cause a decrease in the overall density of the formulation. This enables the formulation to float upon the gastric media [39]. The highest FLT was observed in the formulation F1 which contained the highest amount of polymer and gum (HPMC 110 mg and gum 75 mg). Conversely, formulation F4 contained the lowest amount of polymer and gum (HPMC 60 mg and gum 50 mg) exhibited the lowest FLT. In the case of TFT, the formulation F2 (containing a lower amount of polymer) floated for around 18 hours in tested media while the formulation F1 (containing a higher amount of the polymer) around 25 hours. Thus, the rapid disintegration of the tablets is evident from formulation F2. It appears that the viscosity of the polymer and the corresponding, amount in the formulation, might be considered to be associated directly with the FLT and TFT of the floating tablets [39]. Nevertheless, it is apparent from the results that when the concentration of gum and polymer increased, both FLT and TFT also increased and vice-versa.

#### 3.3.2. Swelling index

A gradual and uniform swelling index was observed in all the formulations (Table 5). However, the formulation F1 showed the highest swelling index 77.23 ± 1.722% (2 hours) to 139.66 ± 1.44% (12 hours) and formulation F4 showed the lowest swelling index 59.87 ± 1.28% (2 hours) to 110.76 ± 1.33% (12 hours) respectively. Again the concentration of polymers appeared to be directly related to the swelling of the tablets [20, 40].

**Table 5:** Cumulative swelling index (%) of propranolol HCl floating tablets.

Time (h)	F1(± SD)	F2(± SD)	F3(± SD)	F4(± SD)
**2**	77.23 ± 1.722	60.75 ± 2.74	72.55 ± 1.12	59.87 ± 1.28
**4**	86.45 ± 2.26	69.98 ± 1.96	80.76 ± 1.84	67.34 ± 1.45
**6**	98.73 ± 1.07	81.22 ± 1.93	89.18 ± 2.08	79.86 ± 1.56
**8**	110.56 ± 0.85	92.78 ± 1.00	99.13 ± 0.86	89.09 ± 2.01
**10**	122.39 ± 1.06	102.98 ± 2.01	108.48 ± 1.16	103 ± 1.87
**12**	139.66 ± 1.44	114.45 ± 1.32	120.81 ± 3.58	110.76 ± 1.33

### 3.4. In-vitro drug release studies

**Fig 3:**
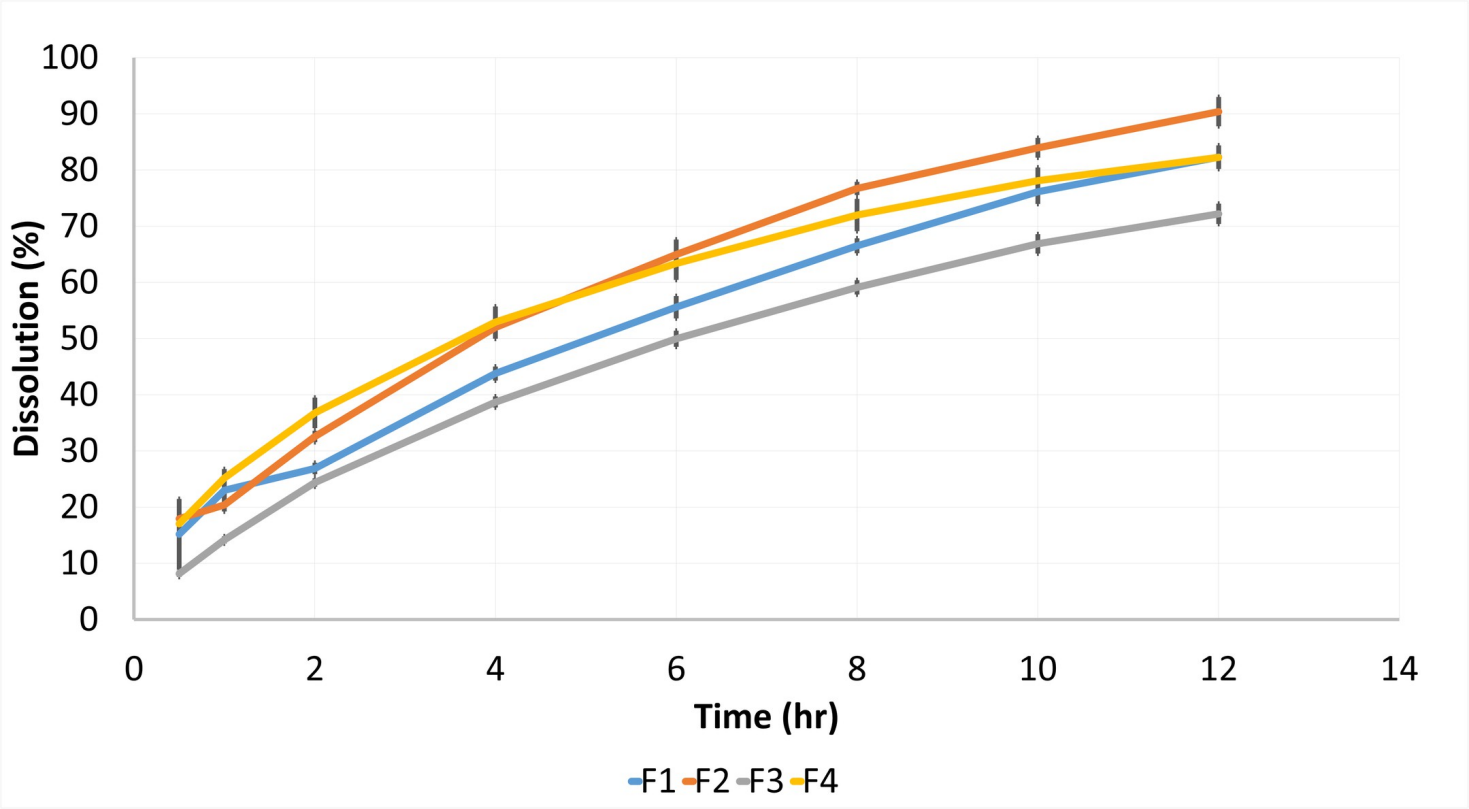
Cumulative percent drug release of trial formulations of propranolol HCl.

The cumulative drug release showed minimum drug release from the formulation F3 followed by F1, F4 and F2 respectively (Fig 3). In the first hour, F1, F2 and F4 formulations appeared to be closer and superimposable except for F3. Then a decline in cumulative drug release was observed in formulations F1 and F3 compared to F2 and F4 and this pattern continued till the last sampling point. The behavior in drug release might be due to the high concentration of polymer in the formulation which formed a strong gel layer around the tablet and thus made the diffusion layer stronger that consequently resulting in a slower release rate. This leads to slow erosion and hindrance in the diffusion of API from matrix tablets (Nigusse et al., 2021). It has been revealed that the formulation F4, containing a lower amount of polymer and gum, showed the initial burst release of Propranolol HCl. A slower drug release was observed in formulation F3. This could be attributed to the binding effect of Avicel PH-102 and the higher concentration of polymer. It is evident from the literature that a higher concentration of polymer is associated with a stronger diffusion layer. This produces a formulation more resistant to erosion and diffusion [41]. However, the cumulative drug release profile of formulation F1 was comparatively better than formulation F3. Nevertheless among all formulations, a gradual and uniform drug release was observed in formulation F4 for 12 hours. It appears that both polymer and gum had produced an appropriate gelling effect that maintained the matrix integrity and resulted slowly with sustained release of the drug till 12 hours.

### 3.5. Release kinetics

**Fig 4:**
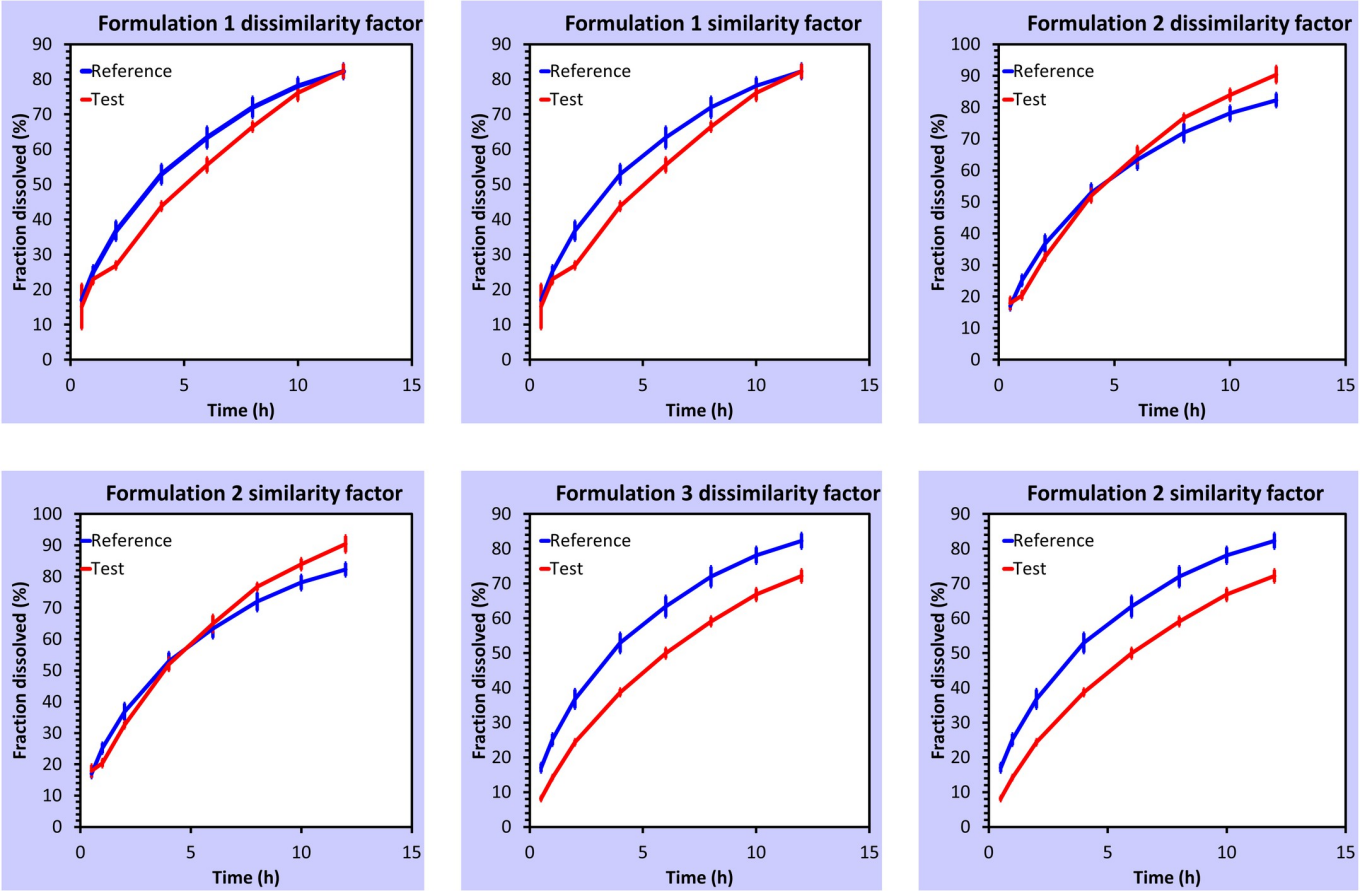
Graphical representation of a pairwise comparison of f1 and f2 factors of propranolol HCl formulations using F4 formulation as reference.

**Fig 5:**
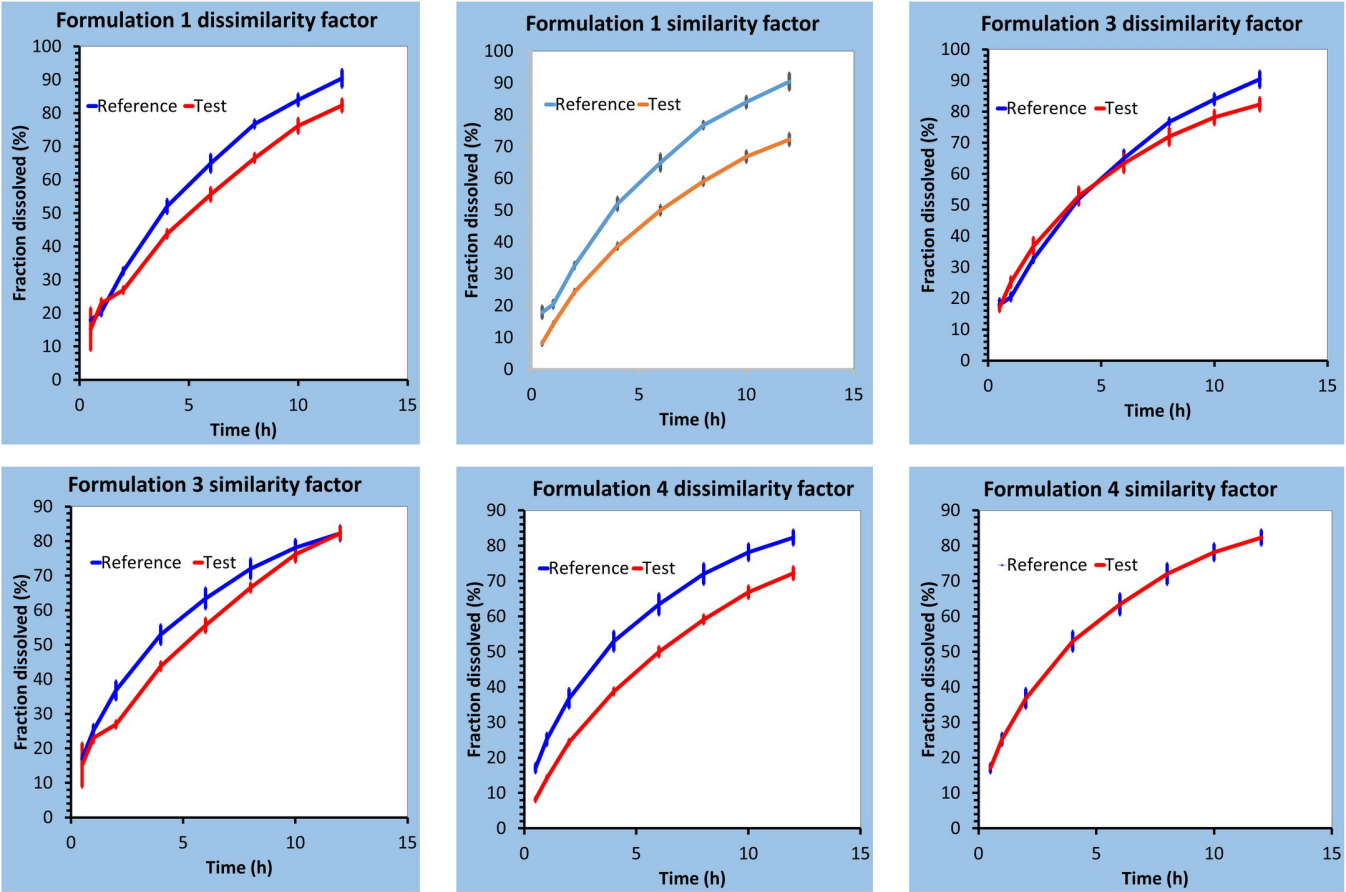
Graphical representation of a pairwise comparison of f1 and f2 factors of propranolol HCl formulations using F2 formulation as reference.

The Weibull model showed the highest determination coefficient (R² = 0.9974–0.9999) followed by Korsemeyer Peppas, Higuchi, First Order, Hixon Crowell and Zero Order respectively (Table 6). Formulations F3 and F4 showed β <1 exhibiting a parabolic curve whilst formulations F1 and F2 showed a sigmoidal curve with β > 1 [20]. The diffusion and erosion of matrix tablets upon hydration were well explained by the Korsmeyer Peppas model. The ‘n’ specifically determined the drug release mechanism from the polymeric system [28]. Only formulation F4 followed Fickian diffusion drug release (n ≤ 0.5) while the rest of the formulations F1, F2 and F3 showed non-Fickian drug release (n >0.5 but<1.0) [42, 43]. Moreover, the dissolution data were analyzed by *f1* and *f2* factors. The factors were applied using formulation F2 or F4 as a reference due to their comparable drug release profile as attributed by paired t-test (p<0.05) to whom all other formulations were compared. The formulations were accepted and rejected based on statistical acceptance criteria i.e., the mean values (Mean R and Mean T). The range 0 to 15 for *f1* and 50 to 100 for *f2* were considered acceptable as recommended for pairwise comparison [44, 45]. Based on these criteria, the formulations F1 and F2 were found similar to F4, or the formulations F1 and F4 were found similar to F2 while the formulation F3 was found dissimilar in both the cases (Figs 4 and 5).

**Table 6:** Invitro kinetic models of propranolol HCl floating tablets.

Formulations	Zero Order		First Order		Higuchi		Korsmeyer Peppas			Hixon Crowell		Weibull Model	
	R^2^	K_o_	R^2^	K_1_	R^2^	K_H_	R^2^	K_KP_	n	R^2^	K_HC_	R^2^	β
**F1**	0.8083	7.87	0.9621	0.145	0.9874	23.209	0.995	20.354	0.565	0.9397	0.04	0.9974	1.362
**F2**	0.7805	8.846	0.9821	0.188	0.9891	26.163	0.9941	23.604	0.551	0.9624	0.051	0.9989	1.277
**F3**	0.8749	6.926	0.9889	0.115	0.9729	20.3	0.9956	15.842	0.623	0.9689	0.033	0.9998	0.877
**F4**	0.5833	8.334	0.9345	0.176	0.9914	24.968	0.9941	26.631	0.468	0.8699	0.047	0.9999	0.782

### 3.6. Optimization of propranolol floating tablets by multiple regression method

Multiple regression (MR) is considered an effective tool for generating prediction equations that provide suitable measurement [33]. The responses of *Prunus domestica* gum and HPMC K4M on the swelling index and drug dissolution obtained in the present study were subjected to MR analysis to find the relationship between the independent and the dependent variables and to optimize the best propranolol HCl floating tablet.

**Fig 6:**
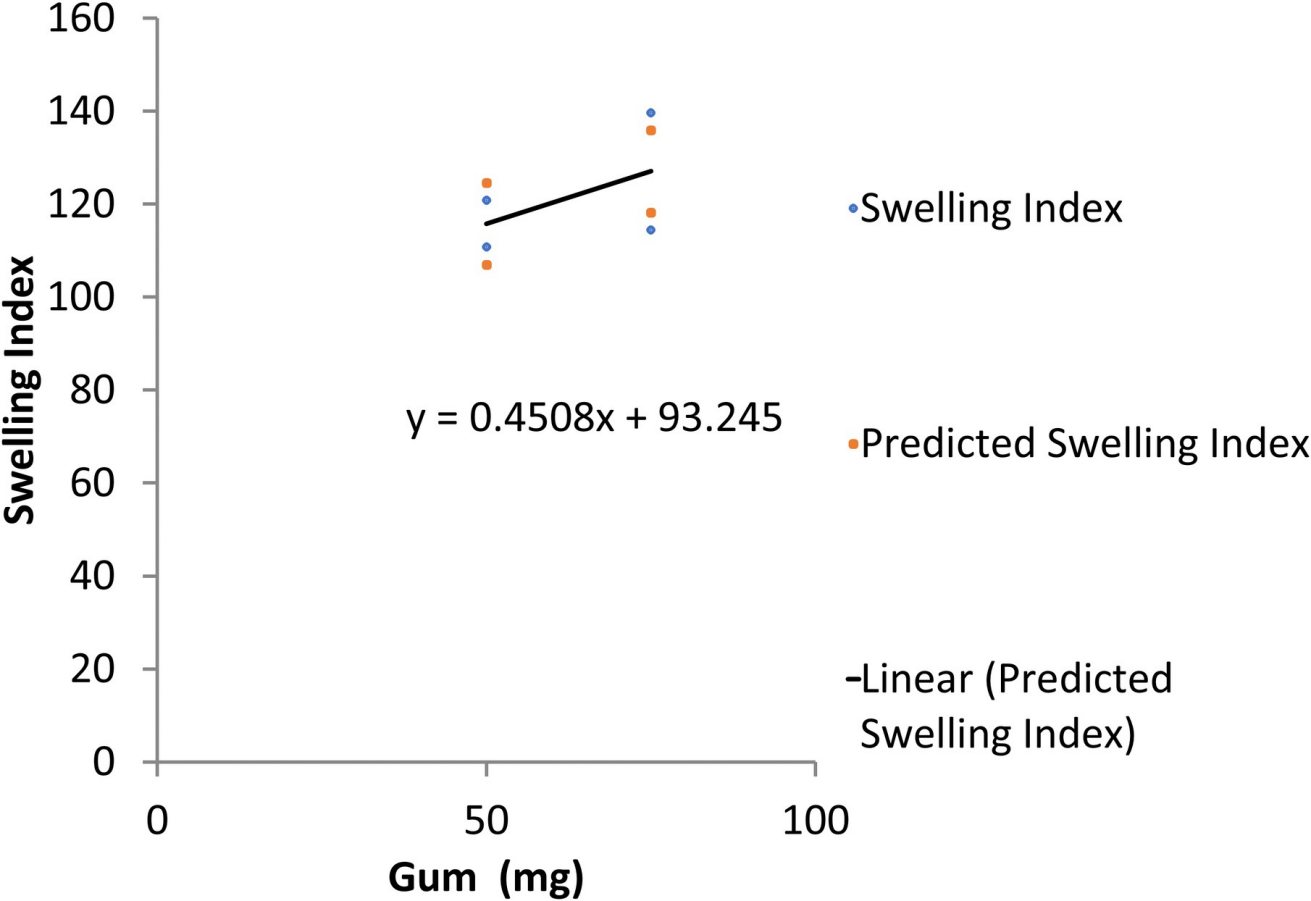
Line fit plot for the response of swelling index with Prunus domestica gum.

**Fig 7:**
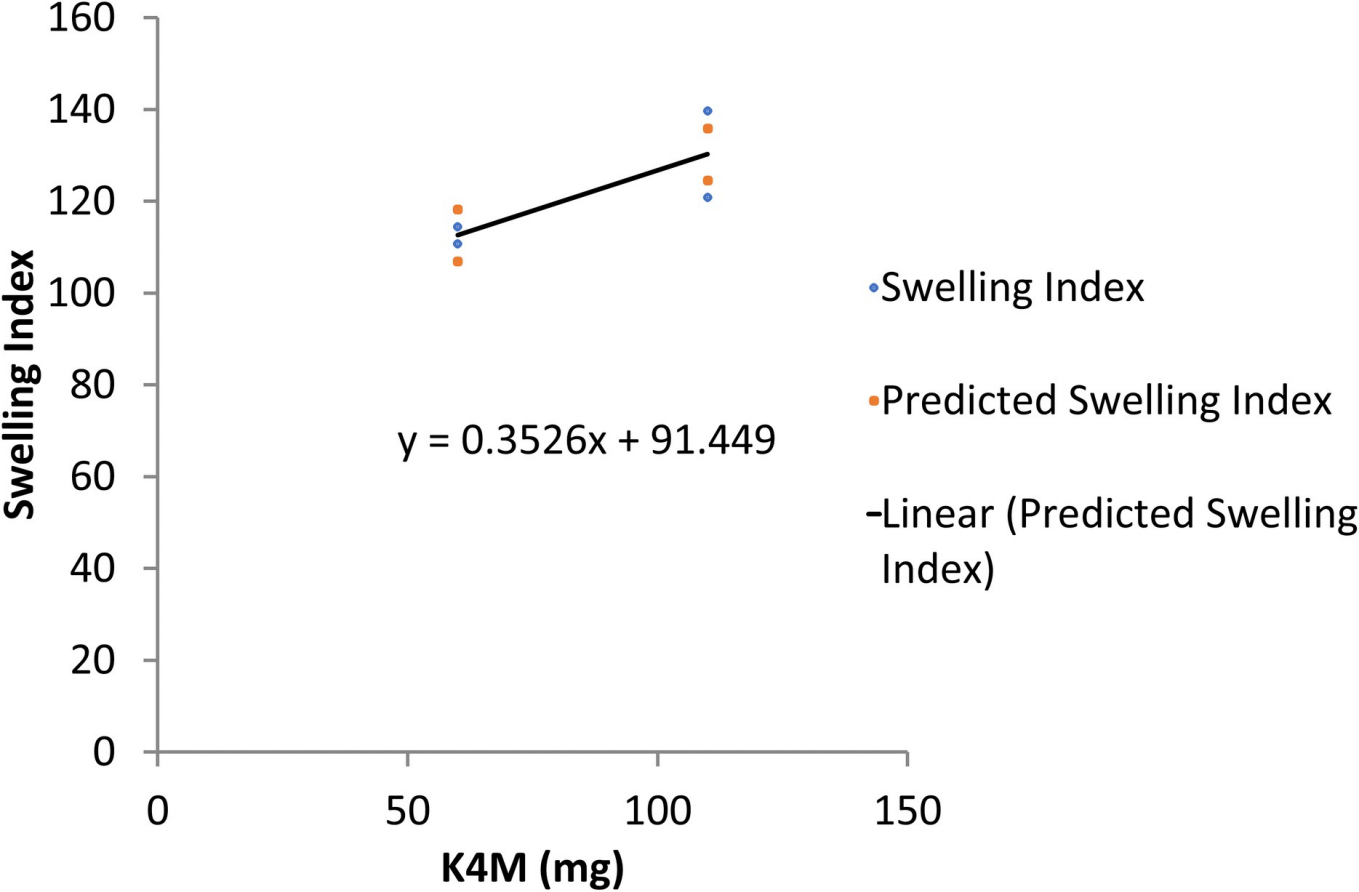
Line fit plot for the response of swelling index with HPMC K4M.

The regression statistics showed an R^2^ of 0.884 for the swelling index after 12 hours of floating of the propranolol HCl formulation. The data was analyzed by ANOVA which detected a significant difference in the swelling index of all trial formulations (p<0.05). Both gum and polymer resulted in a positive value of regression coefficient i.e. 0.45 and 0.35 respectively. It indicates a direct relationship between gum/polymer and swelling index. It means that when the concentration of either gum/polymer increased, the swelling index also increased as verified and depicted in line fit plots (Figs 6 and 7).

**Fig 8:**
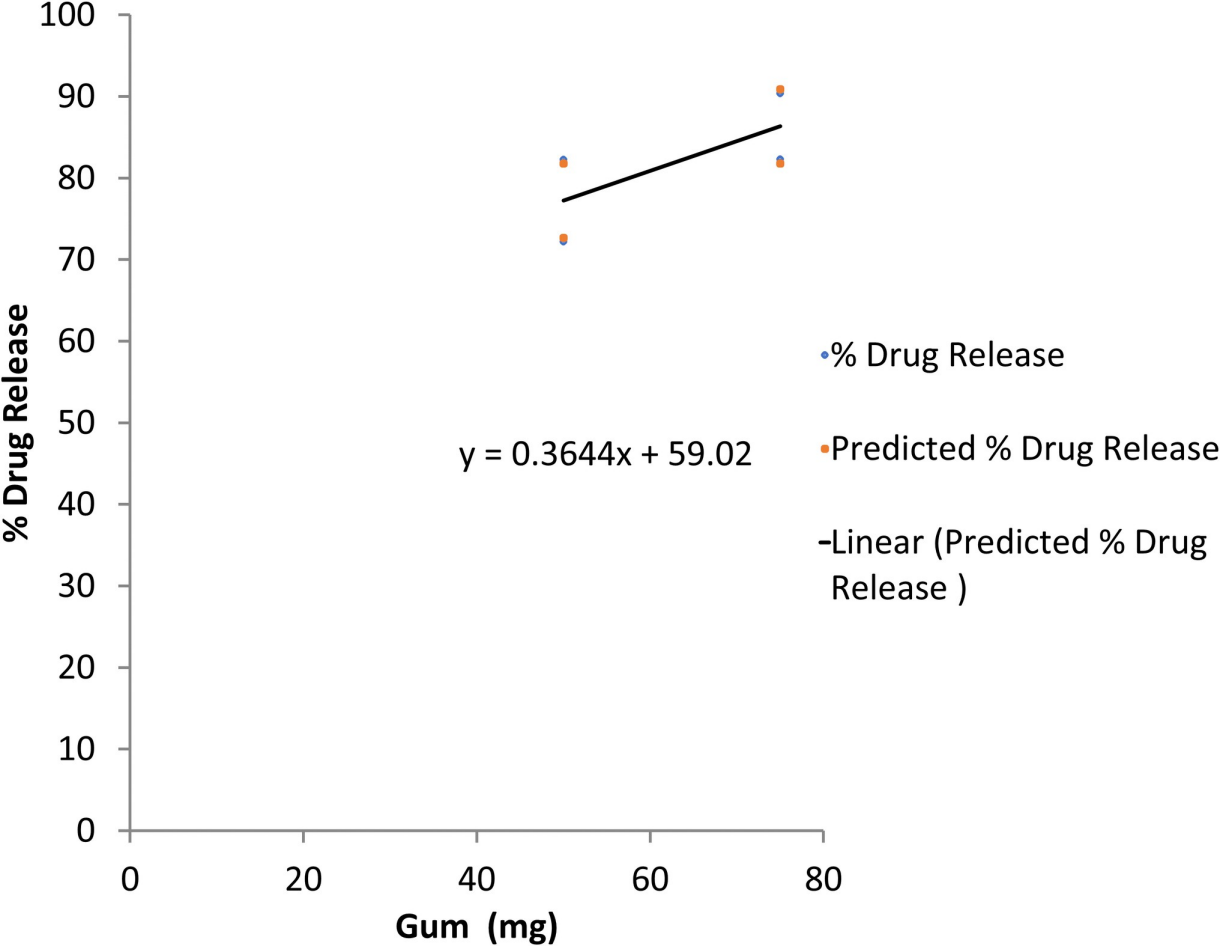
Line fit plot for the response of percent drug dissolution with Prunus domestica gum.

**Fig 9:**
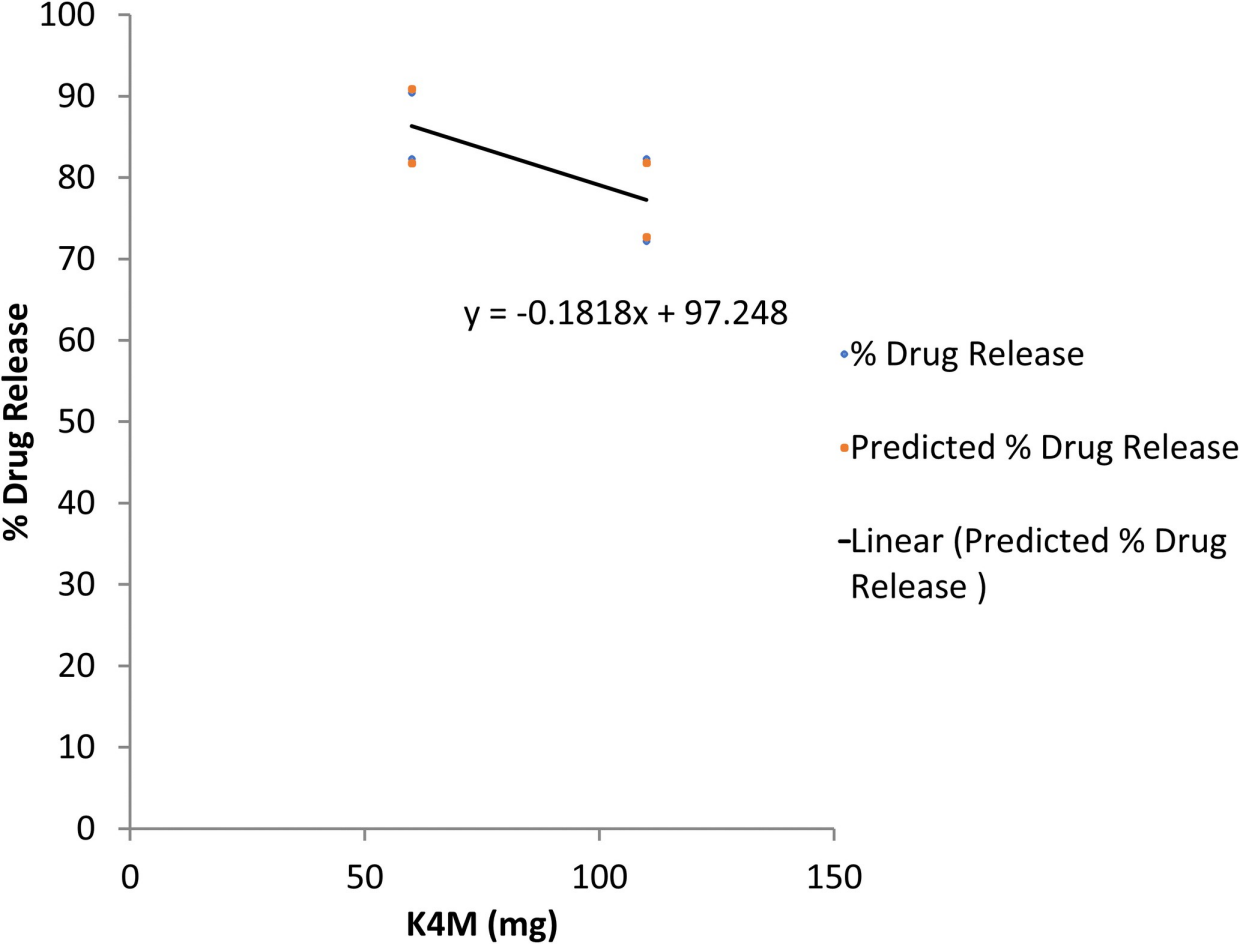
Line fit plot for the response of percent drug dissolution with HPMC K4M.

The regression statistics showed an R^2^ of 0.994 for cumulative drug dissolution of propranolol HCl after 12 hours of sampling time. A higher value of R^2^ indicates a close relationship among the dissolution profiles of all trial formulations. However, when analyzed statistically, a significant difference in the dissolution profiles was detected by ANOVA (p<0.05). Furthermore, the regression coefficients were 0.364 for gum and -0.1820 for HPMC K4M respectively. Contrary to the swelling index where both variables were positive, here we get an opposite effect of both variables on the drug dissolution profile. The positive value of gum showed a direct relationship of gum concentration on the dissolution whilst the negative value of HPMC K4M indicated an inverse relationship between the polymer concentration and dissolution. Both relationships were further confirmed by line fit plots (Figs 8 and 9). Although the formulations F2 and F4 showed comparable characteristics, the formulation F4 might be declared as the best formulation based on low consumption of gum and polymer (making it cost-effective), analysis of multiple regression and other desirable physicochemical attributes of FDDS as described earlier.

## 4. Conclusions

The influence of *Prunus domestica* gum as a matrix former in floating tablets was studied for the first time. A floating time of more than 25 hours was successfully obtained using the gas formation technique. The drug release mechanism showed non-Fickian diffusion except for the formulation F4 which confirmed a Fickian diffusion. The formulation F4 could be considered the best formulation because it contained a lower concentration of gum and polymer (cost-effective) and met all the desirable attributes targeted in the present study. Thus *Prunus domestica* gum appeared to be a suitable candidate for the formulation of FDDS. The formulation will help minimize the limitations related to oral therapy as well as reduce therapy cost, improves patient compliance and make production easy due to the high natural availability, low cost and nontoxic nature of the excipient. Its role alone or in combination with other synthetic and natural polymers should be explored in future studies. To further determine its usefulness as a new matrix former in the formulations of FDDS and other controlled release formulations.
